# Paternity share predicts sons’ fetal testosterone

**DOI:** 10.1038/s41598-023-42718-6

**Published:** 2023-10-04

**Authors:** Ruth Fishman, Lee Koren, Rachel Ben-Shlomo, Uri Shanas, Yoni Vortman

**Affiliations:** 1 Department of Brain Sciences, Weizmann Institute of Science, 76100 Rehovot, Israel; 2 The Mina and Everard Goodman Faculty of Life Sciences, Bar-Ilan University, 5290002 Ramat-Gan, Israel; 3 Faculty of Natural Sciences, University of Haifa—Oranim, 3600600 Tivon, Israel; 4 Hula Research Center, Department of Animal Sciences, Tel-Hai College, 1220800 Upper Galilee, Israel; 5 MIGAL—Galilee Research Institute, 11016 Kiryat Shmona, Israel

**Keywords:** Behavioural ecology, Invasive species, Reproductive biology, Sexual selection

## Abstract

Multiple paternity is common in many species. While its benefits for males are obvious, for females they are less clear. Female indirect benefits may include acquiring ‘good genes’ for offspring or increasing litter genetic diversity. The nutria (*Myocastor coypus*) is a successful invasive species. In its native habitat, it is polygynous, with larger and more aggressive males monopolizing paternity. Here, using culled nutria we genetically examined multiple paternity in-utero and found a high incidence of multiple paternity and maintenance of the number of fathers throughout gestation. Moreover, male fetuses sired by the prominent male have higher testosterone levels. Despite being retained, male fetuses of ‘rare’ fathers, siring commonly only one of the fetuses in the litter, have lower testosterone levels. Considering the reproductive skew of nutria males, if females are selected for sons with higher future reproductive success, low testosterone male fetuses are expected to be selected against. A possible ultimate explanation for maintaining multiple paternity could be that nutria females select for litter genetic diversity e.g., a bet-hedging strategy, even at the possible cost of reducing the reproductive success of some of their sons. Reproductive strategies that maintain genetic diversity may be especially beneficial for invasive species, as they often invade through a genetic bottleneck.

## Introduction

Mating with multiple male partners is common throughout the animal kingdom, from insects to mammals, and is relatively common among mammals, occurring in at least 133 species^1,2^. While mating with more than one partner may enhance male reproductive success, female reproductive success is usually limited by the number of ova produced or offspring raised^3^; thus, the benefits of multiple male partners are less obvious^3^. Furthermore, mating with multiple partners entails costs, including increased exposure to sexually transmitted diseases and increased vulnerability to predation and injuries from potential mating partners, as well as the dangers and time and energy costs accompanying mate-searching and interactions with aggressive males^2,4^. The maintenance of this trait in spite of its costs suggests some benefits^2^. In species in which the males provide no parental care, females may obtain direct benefits such as food gifts, avoidance of sexual harassment and infanticide, fertility insurance^3^ and increased litter size (reviewed in^5^). The genetic benefits of multiple paternity, however, are less straightforward. On the one hand, sexual selection should drive females to select for the highest quality male and enhance offspring genetic quality^6^. On the other hand, multiple paternity may also impose genetic benefits for the females^2,7,8^. These may include inbreeding avoidance^9^, enhanced genetic diversity for their offspring (e.g.,^2,8^) or of the litter (i.e., bet-hedging^10^), or acquisition of ‘good genes’^2,4^, thus increasing offspring fitness (i.e., survival and/or reproductive success (e.g.,^2^).

The steroid hormone testosterone (T) has an important role in mediating male reproductive trade-offs^11,12^. It increases male reproductive success by promoting courtship and sexual behaviors, secondary sexual characters, sperm production and territorial aggression, while often simultaneously bearing a cost by suppressing traits such as immune function and parental care^11–13^. Thus, in species in which territorial males benefit from a high reproductive skew the association between T and reproductive success may be more pronounced. In such species, higher T offspring may contribute to their mothers' fitness (e.g.,^14^).

We examined the incidence of multiple paternity within feral nutria (*Myocastor coypus*) and the possible benefits of multiple paternity to females. The nutria, a semi-aquatic mammal, is one of the fourteen mammalian species included in the ISSG list of the world's 100 worst invasive species^15^. Native to South America, it has been introduced as a furbearer into most continents and major temperate regions except Australia, Antarctica, and New Zealand^16^. Well-adjusting and regulated only by severe winters, nutria populations have reached high numbers in many areas^16^. Damage by nutria to water control structures, crops, and marsh systems and its consideration as a disease vector have led to extensive eradication and control efforts worldwide^16^.

We used a sample of pregnant nutria carcasses attained from culling efforts in Agmon Hula Park in Israel to investigate multiple paternity in utero, fetal morphological features and fetal hair T levels. We examined whether multiple paternity occurs in this species, what characterizes the fetuses sired by different fathers and what fitness related benefits may be associated with it in the Agmon Hula Park population.

According to the ‘good genes’ hypothesis, females can benefit their offspring via the inheritance of genes that may enhance the viability of the offspring or increase their sexual attractiveness (e.g.,^2^). The nutria group structure comprises juveniles, several adult and sub-adult males and females, and one dominant male. The dominant male is significantly larger than the other adult males of the group, differentially more aggressive, and has greater reproductive success^17,18^. Thus, this social structure may imply a fitness benefit for the mother fertilized by the territorial male (even if it had been previously fertilized by a subordinate male), selecting for sons with potentially higher reproductive success. Moreover, fetal resorptions and abortions are prevalent in nutria^19–22^. They have been shown to selectively abort entire litters based on sex ratios and resource availability^20^. Furthermore, intrauterine growth retardation, which implies future fetal resorption or abortion, is significantly more prevalent in male fetuses^23^. Male fetuses tend to be longer and heavier and are apparently the more "costly" sex to produce^20,23^. The selective fetal resorption may enable nutria to have a post-copulation selection mechanism, further increasing the force of sexual selection.

On the other hand, if there are alternative genetic mating strategies, (e.g.,^24,25^) negative frequency dependent selection may maintain the rare morph, maintaining genetic polymorphism. Increased genetic diversity is expected to be particularly adaptive for non-native species^26^, as they face the challenges of adaptation to new environments and a decrease of genetic diversity due to the founder effect and genetic bottlenecks^27,28^. Here we examine whether multiple paternity occur within litter, in the nutria. We further examine the in utero characteristics of the fetuses sired by the main or rare father in relation to size and testosterone levels.

## Materials and methods

### Sample collection

All animals were collected in the Agmon Hula Park, in Northern Israel (33° 10′ N 35° 60′ E), as a secondary use of culling efforts by the authorities at the park in 2013–2019. No live animals were used. Permits are not needed for collecting carcasses because nutrias are an invasive species, not protected by Israeli law. Our main sample comprised 58 litters that included 329 fetuses (mean litter size 5.7 ± 0.08 S.E., range 2–9 fetuses), from a gestational age of 52–138 days, where fetuses show differentiation of corporal regions. Gestational age was estimated using of Newson's formula^21^ cross-validated with multiple fetal morphometric measurements^23^. Sexing of the fetuses was done by anatomical and molecular tools as described in^23^. The 58 mothers and their fetuses were genotyped as described in the following section.

In addition to the main sample, 14 adult male carcasses (4.1–7.1 kg, mean weight 5.69 ± 1.06 kg) were collected. The males served solely to examine the relationship between body size and T levels in adult males and compare adult male and female T. These males were not part of the genetic analysis (siblicity analysis). Adult male and female nutria were weighed using a spring scale (Pesola, Switzerland, 10 kg capacity, 100 g division); length measurements were done using a standard measuring tape and included total length, right hind foot length and tail length. Skull width was measured using a caliper. Few of the specimens were physically damaged during culling, most notably in the skull region, reducing the sample size for this measurement. However, we included this measurement as it has been related to T in some species (e.g.,^29,30^). The fetuses were removed from the uterus and weighed using an electronic balance to the nearest 0.01 mg (Precisa, Switzerland, BJ610C, d = 0.01 g). A standard measuring tape was used to measure fetal length from crown to rump, which could be measured accurately in fetuses from the estimated gestational age of ~ 84 days, and length of right hind foot, measured from the estimated gestational age of ~ 80 days.

Fetuses distinctly smaller than their siblings were observed in some litters. Fetuses reaching only 80% of the litter’s mean weight and lower than the 25th percentile were defined as growth-retarded (Supplemental Information B, Figure SI-B1), with the potential to be resorbed or expelled as the pregnancy progresses.

### Molecular methods

The molecular analysis was performed on the mothers and their fetuses. Tail clippings were excised from 329 fetuses belonging to 58 litters, and ear clippings were excised from all mothers (n = 58). Tissues were preserved in 70% ethanol and refrigerated at -80 °C until processing. Total genomic DNA was isolated from 20 mg tissue and extracted using Geneaid gSYNC DNA Extraction Kit GS100 (Geneaid Biotech Ltd., Taiwan). DNA concentration and cleanness were checked by NanoDrop (Thermo Nanodrop 2000) and diluted as necessary. PCR reactions were performed on the 7 most variable of the 27 microsatellites (Supplemental Information A, Table SI-A1) developed by^31^. PCR reactions contained 7 μL of Taq Ready Mix (Hy Laboratories Ltd, Israel), 4 μL of DDW, 1 μL of 10 μM dye-labeled forward primer (Rhenium limited, Modi'in, Israel), 1 μL of 10 μM reverse primer (Rhenium limited, Modi'in, Israel) and 1 μL of DNA for the markers McoD69, McoD215, McoD60, McoD214, McoA02 or McoD217. For the marker McoD10, 2 μL of every primer was used, and accordingly, 2 μL of DDW. All PCR reactions were performed in the following program: initial denaturing at 94 °C for 2 min; 35 cycles of 94 °C for 30 s, 56 °C for 30 s, 72 °C for 1 min; and a final extension at 72 °C for 10 min.

### Genotyping

Fragment analysis was performed using ABI 3100 Genetic Analyzer. We used Peak Scanner Software v1.0 to determine allelic designations. Alleles that were not clear enough for designation were coded as missing data. We sporadically reran the PCR and the genotyping procedures for various samples to validate the genotyping. A small percentage of the genotypes (0.027) were missing from the final data set. As fetuses were extracted from their mother's carcass, we had the benefit of knowing (with 100% certainty) the assignment of mothers to their offspring. We used this ‘ground truth’ to conduct maternity analysis and resolve mismatches. Allele frequency and maternity analyses were performed using Cervus version 3.0.7 software^32^. Full and half-sib analysis, the minimum number of fathers and statistical paternity assignment were performed using Colony version 2.0.6.5 software^33^, with 4 repeats of > 6 million iterations. To assign sibship Colony implements a maximum likelihood approach, using genotypes of multilocus codominant markers^33^. We further analyzed genetic diversity differences between single and multiple paternity litters, including observed heterozygosity, and mean number of alleles per locus using GenAlEx version 6.5b4 software^34^.

### Testosterone measurements

T in the hair of the mothers and the adult males was extracted and quantified as detailed in^23^. Fetuses' T extraction and quantification were performed as detailed in^35^. The quantification was done using a commercial ELISA kit (Salimetrics Europe, Newmarket, UK) for T. Intra-assay CV was 1.96% for six repeats on the same plate, and inter-assay CV was 8.12% across four plates.

Hair follicles start to appear on nutria fetuses at 85–90 days of gestation. However, most litters do not have a sufficient amount of hair for T quantification until approximately 110 days of gestation. Thus, for the hair T level analysis, our sample constituted fetuses from the gestational age of 110–138 days. As expected, there was no association between their T levels and estimated gestational age (N = 91, F_1,14_ = 0.03, *P* = 0.86)^35^, and in accordance with our previous study, male fetuses' T levels were not related to their intrauterine position (N = 44, F_1,40.08_ = 0.87, *P* = 0.36)^35^. We used 91 fetuses (44 males and 47 females) belonging to 16 litters that had been genotyped and analyzed for siblicity as described above.

### Statistical analyses

For analyses relating to multiple paternity, the variables used were the number of assigned fathers (1–4 fathers per litter) and a more conservative analysis, 'paternity group': A–having no multiple paternity (the entire litter is assigned to a single father), and B–having more than one assigned father, for analyses relating to the mother and to the entire litter. Differences in mean number of alleles per locus as well as correlation between number of fathers and mean number of alleles per locus were examined using *t*-test, assuming unequal variance or Spearman's correlation, respectively. To examine the association to individual fetuses, 'father's proportion in litter' refers to the ratio of fetuses in the litter assigned to a certain father divided by the litter size. Since the nutria is polygynous (and potentially polygamous), the terms 'within-pair' and 'extra-pair' father were replaced by the terms 'single father' for litters with no multiple paternity, 'main father' for the father with the highest incidence in the litter and 'rare father' for those with lower incidence, creating the variable 'father's incidence group in a litter'. There were three litters in which two fathers sired an equal number of fetuses (in addition to a third father, which sired only one fetus). In those cases, both high-siring fathers were considered as “main”. Litter size was examined in relation to the number of assigned fathers and the paternity group using linear regression and *t*-test, respectively. The number of assigned fathers in relation to gestational age was examined using linear regression.

The segregation of fetuses sired by a main or rare father in the uterine horns was examined in the multiple paternity litters by comparing the number of occurrences of fetuses sired by a father in one or both uterine horns. We excluded one litter in which the uterus had been damaged and 3 unilateral pregnancies in which the fetuses resided only in one uterine horn.

Linear mixed models were used to examine fetal weight and length in relation to the fetuses' assigned father's proportion in a litter (the predicting variable) in a model including gestational age and maternal ID as a random factor to account for the fetal uterine environment. The sex ratio of each assigned father was examined using a generalized linear model with binomial distribution, in which the father's proportion in a litter was the predicative variable and the dependent variable was the father's sex ratio (number of males sired by the father divided by the father occurrence in the litter).

Fetal hair T levels were log-transformed to achieve a normal distribution and examined both in relation to their father's proportion in the litter and in relation to their father's incidence group in linear mixed models including maternal ID as a random factor, where sex and father's proportion in the litter and, separately, father's incidence group were the model effects, and then examined again separately for each of the sexes. Nutria fetuses T levels significantly relate to their sex (Fishman, 2019) as observed also in this sample (N = 91, sex: F_1,78.07_ = 5.05, *P* = 0.027).

The associations between the number of assigned fathers in the litter and the mother's morphological measurements—total length, hind foot length and tail length—as well as Johnson SI transformed maternal T levels were examined using linear regressions. Linear regressions were also used to examine maternal T levels in relation to their total length, skull width, tail length and hind foot length. To examine maternal T levels in relation to their weight, we constructed a model with maternal weight, gestational age and litter size as model effects. Adult male T levels were Johnson SI transformed to achieve normal distribution and examined in relation to their weight, total length, skull width, tail length and hind foot length using linear regressions. Comparing adult male and female T levels was performed using *t*-test assuming unequal variance. Model fitting was performed in JMP (version 13, SAS Inc.).

## Results

### Within-litter multiple paternity

Following genetic siblicity analysis we found multiple paternity in 34 of the 58 litters (58.6%), i.e., only 41.4% of the litters contained full sibs related to a single father (Supplemental Information A, Table SI-A2). In multiple paternity litters, number of fathers ranged from 2 to 4 fathers, and the mean proportion in the litter of the main fathers was 0.62 ± 0.03 S.E. and of rare fathers 0.24 ± 0.01 S.E.

Multiple paternity litters, as expected, had significantly higher mean number of alleles per locus (multiple paternity litters: 2.62 ± 0.29 S.E.; single paternity litters: 2.38 ± 0.23 S.E., t = 3.50, *df* = 54.96, *P* = 0.0009). This was also portrayed in a significant positive correlation between number of fathers and mean number of alleles per locus (R_Spearman_ = 0.53, N = 58, *P* < 0.0001). However, there were no significant differences in observed heterozygosity between single and multiple paternity litters (Ho = 0.611 ± 0.058 and 0.624 ± 0.066 respectively).

No relationship was found between the number of assigned fathers and litter size (N = 58, R^2^ = 0.006, *P* = 0.57), nor when comparing paternity groups i.e., litters with a single father and those with multiple fathers (N = 58, t_56_ = 0.08, *P* = 0.94). There was also no decline in the number of fathers as the pregnancy progressed (N = 58, R^2^ = 0.017, *P* = 0.33).

### Lack of segregation of sired fetuses in the uterine horns

We did not identify a specific pattern of segregation of fetuses sired by the main father in the uterine horns. Excluding unilateral uterine horn pregnancies, in 23 litters out of 30 multiple paternity litters, the fetuses sired by the main father resided in both of the uterine horns. The same lack of segregation by uterine horn appeared also in relation to fetuses sired by the rare father. 35 of the rare fathers had sired only one fetus in the litter. Out of 13 assigned rare fathers that sired more than a single fetus (one rare father sired 3 fetuses and the rest 2 fetuses), 7 sired fetuses in both of the uterine horns, and 6 in a single uterine horn (4 in the right uterine horn and 2 in the left).

### Paternity and fetal characteristics

The sex of the fetuses was not related to their assigned fathers' proportion in the litter (N = 101 fathers, χ^2^_1_ = 0.48, *P* = 0.49). Fathers' proportion in the litter did not predict the fetuses weight (N = 316, gestational age: F_1,56.14_ = 329.78, *P* < 0.0001; father proportion in the litter: F_1,295.2_ = 0.26, *P* = 0.61). Fathers' proportion in the litter also did not predict fetal length measurements: crown-rump length (N = 155, gestational age: F_1,31.62_ = 1034.57, *P* < 0.0001; father proportion in the litter: F_1,102_ = 0.64, *P* = 0.43) and right hind foot length (N = 186, gestational age: F_1,29.88_ = 3550.51, *P* < 0.0001; father proportion in the litter: F_1,80.72_ = 1.69, *P* = 0.2).

**Figure 1:**
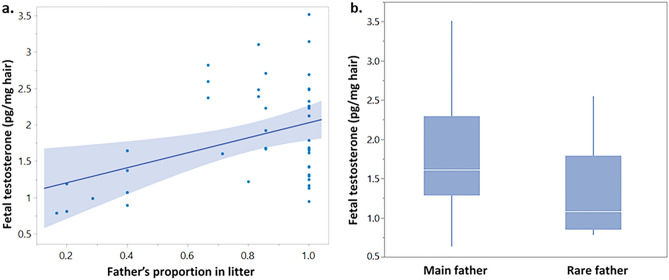
Male fetal T levels: (**a**, **b**) T levels of male fetuses of *M. coypus*. (**a**) Male fetuses’ T levels in relation to their father’s proportion in the litter, this relationship is significant (*P* = 0.0054). (**b**) Box plot of male fetuses' T levels in relation to their assignment to rare father versus main and single fathers (grouped into main fathers group); the two groups are significantly different (*P* = 0.0005). The mother's identity was incorporated as a random factor in the statistical model (see text for detailed statistics).

Nutria fetuses T levels are related to their sex (N = 91, sex: F_1,78.07_ = 5.05, *P* = 0.027). A positive association was found between the father’s proportion in the litter and fetal T levels, incorporating fetal sex (N = 91, sex: F_1,77.62_ = 4.48, *P* = 0.036; father’s proportion in litter: F_1,82.25_ = 4.44, *P* = 0.038), i.e., fetuses sired by the father that sired most of the fetuses had higher T levels. Since male and female fetuses differ significantly in their T levels (see also Fishman et al.^35^), the association between the father's proportion and fetal T levels was also examined for each sex separately. The T levels of female fetuses were not associated with their father’s proportion in the litter (N = 47, F_1,44.68_ = 0.003, *P* = 0.95), while the association for male fetuses was highly significant (N = 44, F_1,29.74_ = 9.02, *P* = 0.0054) (Fig. 1a). Further examination of this association in male fetuses in relation to single/main/rare fathers showed the same result (N = 44, F_2,22.79_ = 7.33, *P* = 0.0035). The Tukey HSD test for all pairwise differences showed that while fetuses related to main or single fathers have significantly higher T than fetuses related to rare fathers, there is no difference between fetuses related to the single or the main father. Thus, fetuses related to single and main fathers were grouped together and their T levels compared to those of fetuses related to the rare father, showing that male fetuses related to rare fathers have significantly lower T levels (N = 44, F_1,35.47_ = 14.73, *P* = 0.0005) (Fig. 1b).

### Multiple paternity and maternal characteristics

The number of fathers in a litter was not associated with the mothers’ morphological measurements nor with maternal hair T levels (total length: N = 57, R^2^ = 0.004, *P* = 0.65; hind foot length: N = 58, R^2^ = 0.003, *P* = 0.69; tail length: N = 57, R^2^ = 0.009, *P* = 0.48; hair T levels: N = 55, R^2^ = 0.03, *P* = 0.23). In addition, neither maternal morphometrics nor T levels were related to paternity group, i.e., the probability of carrying a litter related to a single paternity or to a multiple paternity (total length: N = 57, χ^2^ = 0.29, *P* = 0.59; hind foot length: N = 58, χ^2^ = 0.68, *P* = 0.41; tail length: N = 57, χ^2^ = 0.36, *P* = 0.55; T levels: N = 55, χ^2^ = 0.54, *P* = 0.46).

### Hair testosterone levels and adult characteristics

**Figure 2:**
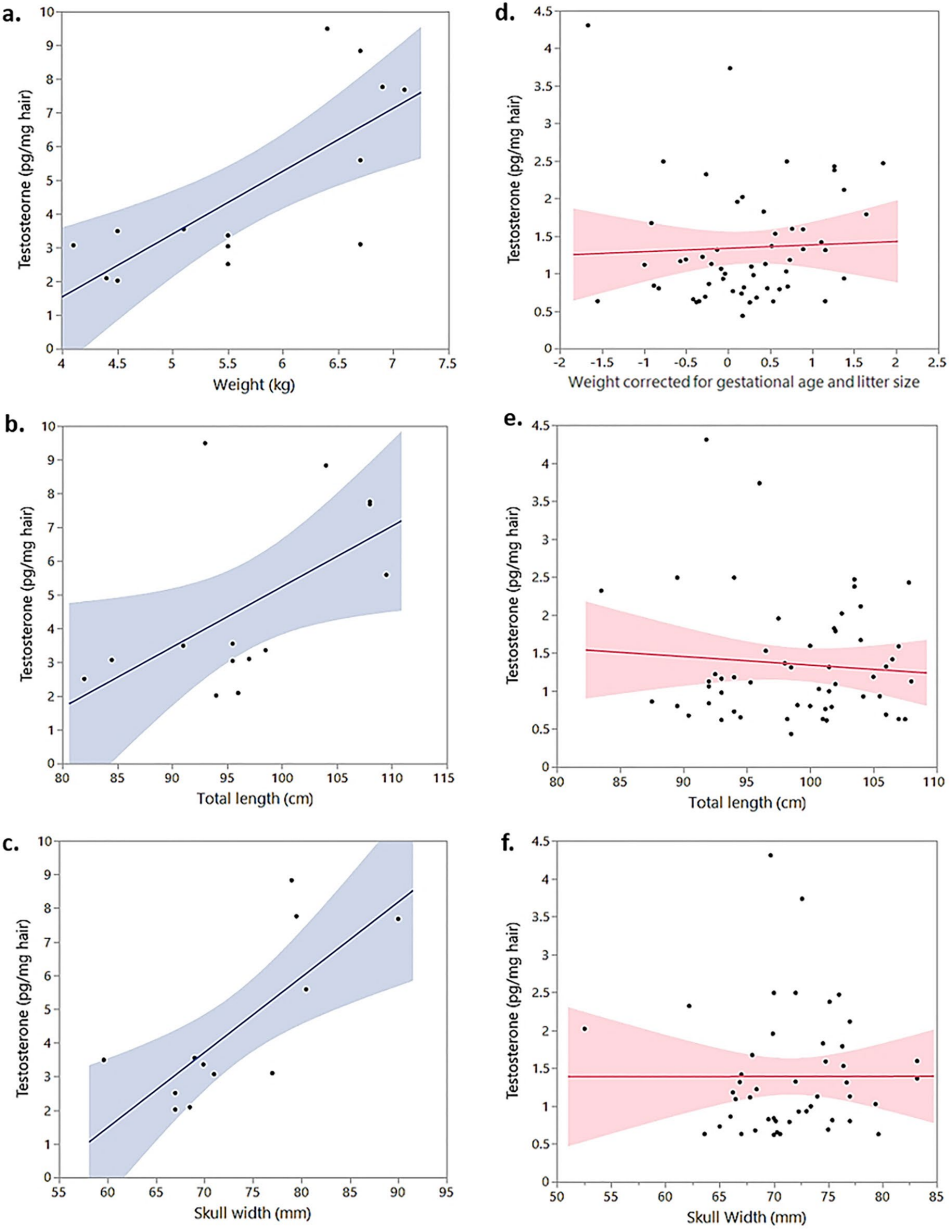
T levels of adult males and females: (**a**–**f**) T levels determined from caracasses of adult males and females of *M. coypus*. (**a**) Adult males' T levels in relation to weight (*P* = 0.0015). (**b**) Adult males' T levels in relation to total length (*P* = 0.04). (**c**) Adult males' T levels in relation to skull width (*P* = 0.015). (**d**) Adult females' T levels in relation to weight corrected for gestational age and litter size, no significant relationship was detected. (**e**) Adult females' T levels in relation to total length, no significant relationship was detected. (**f**) Adult females' T levels in relation to skull width, no significant relationship was detected.

Adult male and female nutria differed significantly in their hair T levels (t_28_ = 8.48, N = 69, *P* < 0.0001). T levels of adult males were positively correlated with various measures of body size (weight: N = 14, R^2^ = 0.58, *P* = 0.0015; total length: N = 14, R^2^ = 0.30, *P* = 0.04; hind foot length: N = 14, R^2^ = 0.38, *P* = 0.018; skull width: N = 12, R^2^ = 0.46, *P* = 0.015; tail length: N = 14, R^2^ = 0.35, *P* = 0.025) (Fig. 2a–c). Adult nutria males' weight no longer correlates with age above ~ 5.5 kg^36^. The significant correlation between body size and T persists also when removing males smaller than 5.5 kg from the analysis to account for any possible effect of age on T levels (weight: N = 9, R^2^ = 0.56, *P* = 0.02; total length: N = 9, R^2^ = 0.44, *P* = 0.049; skull width: N = 7, R^2^ = 0.63, *P* = 0.033; tail length: N = 14, R^2^ = 0.55, *P* = 0.023). Hind foot length however, did not correlate with T in this small sample (N = 9, R^2^ = 0.07, *P* = 0.49).

Adult female nutria body size measurements showed no association with their T levels (total length: N = 55, R^2^ = 0.0003, *P* = 0.90; hind foot length: N = 55, R^2^ = 0.03, *P* = 0.25; skull width: N = 47, R^2^ = 0.003, *P* = 0.74; tail length: N = 55, R^2^ = 0.007, *P* = 0.56; weight corrected for gestational age and litter size: N = 55, model *P* = 0.72) (Fig. 2d–f).

### Fetal growth retardation and paternity

Following the above-mentioned result demonstrating that male fetuses sired by the rare father had lower T levels (Fig. 1), we examined, on a limited sample, whether there is an indication that females selectively absorb low T males or maintain them. We did not find any indication that females selectively absorb fetuses sired by the rare father. On the contrary, out of 7 multiple paternity litters with partly absorbed fetuses only one of the 8 growth-retarded fetuses absorbed was related to a rare father (see Supplemental Information B, Table SI-B1).

## Discussion

Mating with multiple partners may convey costs and benefits to females (e.g.,^2^). In this study, we showed that more than half (i.e., 58.6%) of the nutria litters in the Agmon Hula Park had multiple paternity. We found that the main father in a litter (siring the majority of the fetuses), sired male fetuses with higher T levels (Fig. 1a, b). The number of fathers did not increase with litter size, moreover, potential fetal resorption did not reduce the number of fathers; We thus suggest that multiple paternity in the nutria may impose benefits for the female.

There are a number of possible direct and genetic benefits to multiple paternity. One of the direct benefits is fertility assurance and increased litter size (reviewed in^5^), but we did not find evidence of this.

Fetal morphological features, such as weight and length, did not differ between fetuses related to single, main or rare fathers or to their assigned father's proportion in the litter, suggesting no benefits of fetal morphological development in relation to one type of father. This would not necessarily imply an absence of morphological differences in adulthood^37^. However, we found that **male** fetal T levels were positively associated with the father's proportion in the litter (i.e., male fetuses assigned to higher frequency fathers in the litter had higher T levels). Male fetuses assigned to single or main fathers showed higher T levels compared to fetuses related to rare fathers, this was not the case for female fetuses.

Adult male T levels have been associated with territoriality and dominance (e.g.,^38–41^) size (e.g.,^41–43^) and higher reproductive success (e.g.,^39,40,44^). A sample of adult nutria males also showed a strong positive correlation between the adult males' T levels and size, which did not exist in adult females. In their native habitat, nutria group composition comprises one dominant, differently larger and aggressive adult male and a few considerably less aggressive adult males^17^, potentially leading to a significant reproductive skew between males in the population^17,18^.

Male T levels may change with age, with life phases and in response to environmental and behavioral changes (e.g.,^39,45^), although high repeatability in T levels across time has been demonstrated in some systems (e.g.,^46–48^), but see also^49,50^. Thus, we cannot infer with certainty that nutria males that have higher T levels as fetuses will necessarily have higher T levels as adults. However, a positive correlation between fetal and adult T levels has been found in humans^30^ and an indication of such a correlation also in gerbils^51^. Furthermore, studies show a genetic component of T levels in various species (e.g.,^14,52–56^). Recently, we have examined the heritability of fetal T in the nutria using this study sample. We found that in utero accumulated hair T levels are heritable between parents and offspring of the same sex (i.e., between fathers and sons and mothers and daughters), indicating a genetic component of fetal T (Preprint^57^).

Fetal T levels can also predetermine T levels in adults (e.g.,^58^) and correlate with sexual characteristics in adulthood^59^ via developmental effects. Prenatal T has an organizing action on the tissues mediating mating behavior, as well as behaviors contributing to reproductive success such as aggressiveness, exerting effects that persist through adulthood^60–64^. Intrauterine position effect studies show that higher exposure to T via proximity to males in utero leads to higher fetal T levels, affecting adult sexual morphology^51,59,65^. In males, this fetal exposure to T enhances adult behavior patterns linked to higher reproductive success, such as higher aggressiveness and dominance^65–70^, territorial behavior^70,71^ and dispersal rate^68,70^. Males that develop between two males in utero are more sensitive to T as adults (e.g.,^72–74^), more attractive to females^75^, more effective copulators and have a higher impregnation rate^71,75,76^, heavier testes and seminal vesicles^65,71,77^; overall, presenting higher reproductive success^75^. Thus, although we do not know whether nutria fetal T levels predict their adult levels, male fetuses assigned to main and single fathers have higher T levels, which at least via prenatal effects may impact their reproductive success as adults. This raises an interesting question: assuming that the main father's male fetuses (high T male fetuses) gain greater reproductive success, why would females maintain rare fathers’ male fetuses? This conundrum is similar to the ‘lek paradox’^78^, and accordingly, the evolutionary benefits to the females may be to increase the litter's genetic variation as a tradeoff with the offspring's ‘good genes’ (reviewed in^79^). This speculation is in line with previous nutria field studies (e.g.,^18^), which suggest that nutria have mechanisms to increase genetic diversity, e.g., females pairing with genetically distinct partners or the negative relationship between the level of polygamy and group relatedness (i.e., in highly related groups, there was lower reproductive skew, and more fathers sired the offspring). Túnez et al.^18^ further suggest that the plasticity in reproductive strategies observed in nutria may contribute to its success as an exotic species.

Assuming a link between fetal T levels and future reproductive success (e.g.,^70–72,75,76^), our results suggest that the nutria in Agmon Hula Park may mate multiply as a strategy for increasing litters genetic diversity, even at the possible cost of reducing the reproductive success of some of the sons. This is especially surprising as males are the more “costly” fetuses in the nutria and have a higher tendency for growth retardation (potential future resorption)^23^. With the higher male fetus developmental cost and the potential high reproductive success of large high-quality males, sexual selection should favor maintenance of fetuses related to the main and single fathers. On the other hand, the genetic diversity hypothesis assumes that multiple mating can increase females’ fitness by producing more genetically diverse litters^2,3^. This strategy can serve as a bet-hedging mechanism against stressful conditions and temporal fluctuations in the environment, enabling the survival of at least some of the genotypes within the litter, or maintaining alternative reproductive strategies^2,3,10^. In addition, an analysis on a limited sample, within a constrained time window revealed that growth-retarded fetuses, likely unable to reach full term, belonged almost exclusively to single and main fathers. This finding demonstrates that females do not appear to be selectively absorbing fetuses sired by the rare father (Supplemental Information B, Table SI-B1).

Similarly to other studies, we did not find a significant increase in the observed level of heterozygosity in the multiple paternity litters, however these litters have a mean higher number of alleles (e.g.,^3^). Thus, mating with multiple males does not necessarily produce more genetically diverse offspring but rather more genetically diverse litters, in line with a bet-hedging strategy^3^.

Testosterone has a crucial role in fetuses’ development, regulating growth, maintenance and function of reproductive tissues, gonads and brain^80^. Thus, the demonstrated differences in male fetal T as a function of sire should have implications on the adult nutria male. Since selection is selecting for increasing female’s reproductive success, we can assume that the observed multiple paternity and its relation with fetal T is probably adaptive. We emphasize that the significant relationship we have detected between paternity and fetal T relates to male fetuses, and that in this study we did not find any significant relationship between multiple paternity and female fetuses’ characteristics. It is important to accentuate that females' fitness is influenced not only by the reproductive success of their sons but also by that of their daughters. Selection for multiple paternity may have its effects on female fetuses and their genes. For example, studies on the bank vole (*Myodes glareolus*) have shown that while males from high T families have higher reproductive success, females from low T families demonstrate superior reproductive fitness compared to their counterparts from high T families^81–83^. Thus, mating also with lower T males might contribute to the female's fitness also through its daughters, all the while contributing to conserving genetic diversity^82,83^.

Species with mechanisms that have been selected to increase genetic diversity may be particularly well-equipped to succeed as invasive species, as they need to overcome an inherent founder effect, unknown environmental conditions and strict genetic bottlenecks (e.g.,^84,85^). The tendency of nutria females to mate with multiple males may have been one of the factors enabling this species to become such a successful invader. This suggested link between mating strategies that increase genetic diversity and invasion success may be further examined in other successful invasive species.

### Supplementary Information

Supplementary Information 1.

Supplementary Information 2.

## Data Availability

All morphological, paternity and testosterone related matrices for this manuscript are available at the following repository link: https://data.mendeley.com/datasets/x5zg7zmsxj/1.

## References

[1] Wolff JO, Macdonald DW (2004). Promiscuous females protect their offspring. Trends Ecol. Evol..

[2] Jennions MD, Petrie M (2000). Why do females mate multiply? A review of the genetic benefits. Biol. Rev..

[3] Thonhauser KE, Thoß M, Musolf K, Klaus T, Penn DJ (2014). Multiple paternity in wild house mice (*Mus musculus musculus*): effects on offspring genetic diversity and body mass. Ecol. Evol..

[4] Daly M (1978). The cost of mating. Am. Nat..

[5] Dobson FS, Abebe A, Correia HE, Kasumo C, Zinner B (2018). Multiple paternity and number of offspring in mammals. Proc. R. Soc. B Biol. Sci..

[6] Clutton-Brock T (2007). Sexual selection in males and females. Science.

[7] Simmons LW (2005). The evolution of polyandry: Sperm competition, sperm selection, and offspring viability. Annu. Rev. Ecol. Evol. Syst..

[8] Rubenstein DR (2007). Female extrapair mate choice in a cooperative breeder: Trading sex for help and increasing offspring heterozygosity. Proc. R. Soc. B Biol. Sci..

[9] Tregenza T, Wedell N (2002). Polyandrous females avoid costs of inbreeding. Nature.

[10] Yasui Y (1998). The ‘genetic benefits’ of female multiple mating reconsidered. Trends Ecol. Evol..

[11] Ketterson ED, Nolan V (1992). Hormones and life histories: An integrative approach. Am. Nat..

[12] Hau M (2007). Regulation of male traits by testosterone: Implications for the evolution of vertebrate life histories. BioEssays.

[13] Folstad I, Karter AJ (1992). Parasites, bright males, and the immunocompetence handicap. Am. Nat..

[14] Mills SC (2009). Testosterone-mediated effects on fitness-related phenotypic traits and fitness. Am. Nat..

[15] Lowe, S., Browne, M., Boudjelas, S. & De Poorter, M. *100 of the World’s worst invasive alien species. A selection from the Global Invasive Species Database. The Invasive Species Specialist Group (ISSG) a specialist group of the Species Survival Commission (SSC) of the World Conservation Union (IUCN)* (2004).

[16] Carter J, Leonard BP (2002). A review of the literature on the worldwide distribution, spread of, and efforts to eradicate the coypu (*Myocastor coypus*). Wildl. Soc. Bull..

[17] Guichón ML, Borgnia M, Righi CF, Cassini GH, Cassini MH (2003). Social behavior and group formation in the coypu (*Myocastor coypus*) in the Argentinean pampas. J. Mammal..

[18] Túnez JI (2009). Relatedness and social organization of coypus in the Argentinean pampas. Mol. Ecol..

[19] Chapman JA, Lanning JC, Willner GR, Pursley D (1980). Embryonic development and resorption in feral nutria (*Myocastor coypus*) from Maryland. Mammalia.

[20] Gosling LM (1986). Selective abortion of entire litters in the coypu: Adaptive control of offspring production in relation to quality and sex. Am. Nat..

[21] Newson RM (1966). Reproduction in the feral coypu (*Myocastor coypus*). Symp. Zool. Soc. Lond..

[22] Willner GR, Chapman JA, Pursley D (1979). Reproduction, physiological responses, food habits, and abundance of nutria on Mariland marshes. Wildl. Monogr..

[23] Fishman R, Vortman Y, Shanas U, Koren L (2018). Female-biased sex ratios are associated with higher maternal testosterone levels in nutria (*Myocastor coypus*). Behav. Ecol. Sociobiol..

[24] Lamichhaney S (2015). Structural genomic changes underlie alternative reproductive strategies in the ruff (*Philomachus pugnax*). Nat. Genet..

[25] Dominey WJ (1980). Female mimicry in male bluegill sunfish—A genetic polymorphism?. Nature.

[26] Ellstrand NC, Schierenbeck KA (2000). Hybridization as a stimulus for the evolution of invasiveness in plants?. Proc. Natl. Acad. Sci..

[27] Dlugosch KM, Parker IM (2008). Founding events in species invasions: Genetic variation, adaptive evolution, and the role of multiple introductions. Mol. Ecol..

[28] Franks SJ, Pratt PD, Tsutsui ND (2011). The genetic consequences of a demographic bottleneck in an introduced biological control insect. Conserv. Genet..

[29] Dahinten SL, Pucciarelli HM (1986). Variations in sexual dimorphism in the skulls of rats subjected to malnutrition, castration, and treatment with gonadal hormones. Am. J. Phys. Anthropol..

[30] Whitehouse AJO (2015). Prenatal testosterone exposure is related to sexually dimorphic facial morphology in adulthood. Proc. R. Soc. B Biol. Sci..

[31] Callahan CR, Henderson AP, Eackles MS, King TL (2005). Microsatellite DNA markers for the study of population structure and dynamics in nutria (*Myocastor coypus*). Mol. Ecol. Notes.

[32] Marshall TC, Slate JBKE, Kruuk LEB, Pemberton JM (1998). Statistical confidence for likelihood-based paternity inference in natural populations. Mol. Ecol..

[33] Jones OR, Wang J (2010). COLONY: A program for parentage and sibship inference from multilocus genotype data. Mol. Ecol. Resour..

[34] Peakall R, Smouse PE (2006). GENALEX 6: genetic analysis in Excel. Population genetic software for teaching and research. Mol. Ecol. Notes.

[35] Fishman R, Vortman Y, Shanas U, Koren L (2019). Non-model species deliver a non-model result: Nutria female fetuses neighboring males in utero have lower testosterone. Horm. Behav..

[36] Willner GR, Dixon KR, Chapman JA, Stauffer JR (1980). A model for predicting age-specific body weights of nutria without age determination. J. Appl. Ecol..

[37] Manikkam M (2004). Fetal programming: Prenatal testosterone excess leads to fetal growth retardation and postnatal catch-up growth in sheep. Endocrinology.

[38] Machida T, Yonezawa Y, Noumura T (1981). Age-associated changes in plasma testosterone levels in male mice and their relation to social dominance or subordinance. Horm. Behav..

[39] Clarke FM, Faulkes CG (1998). Hormonal and behavioural correlates of male dominance and reproductive status in captive colonies of the naked mole-rat, Heterocephalus glaber. Proc. R. Soc. B Biol. Sci..

[40] Moss R, Parr R, Lambin X (1994). Effects of testosterone on breeding density, breeding success and survival of red grouse. Proc. R. Soc. B Biol. Sci..

[41] Wickings EJ, Dixson AF (1992). Testicular function, secondary sexual development, and social status in male mandrills (*Mandrillus sphinx*). Physiol. Behav..

[42] Bardin CW, Catterall JF (1981). Testosterone: A major determinant of extragenital sexual dimorphism. Science.

[43] Cox RM, Stenquist DS, Calsbeek R (2009). Testosterone, growth and the evolution of sexual size dimorphism. J. Evol. Biol..

[44] Adkins-Regan E (2005). Hormones and Animal Social Behavior.

[45] Wingfield JC, Hegner RE, Dufty AM, Ball GF (1990). The ‘challenge hypothesis’: theoretical implications for patterns of testosterone secretion, mating systems, and breeding strategies. Am. Nat..

[46] Liening SH, Stanton SJ, Saini EK, Schultheiss OC (2010). Salivary testosterone, cortisol, and progesterone: Two-week stability, interhormone correlations, and effects of time of day, menstrual cycle, and oral contraceptive use on steroid hormone levels. Physiol. Behav..

[47] Kraus S, Krüger O, Guenther A (2020). Zebra finches bi-directionally selected for personality differ in repeatability of corticosterone and testosterone. Horm. Behav..

[48] Mutwill AM (2021). Individuality meets plasticity: Endocrine phenotypes across male dominance rank acquisition in guinea pigs living in a complex social environment. Horm. Behav..

[49] Kralj-Fišer S, Scheiber IBR, Blejec A, Moestl E, Kotrschal K (2007). Individualities in a flock of free-roaming greylag geese: Behavioral and physiological consistency over time and across situations. Horm. Behav..

[50] Pavitt AT, Walling CA, Möstl E, Pemberton JM, Kruuk LEB (2015). Cortisol but not testosterone is repeatable and varies with reproductive effort in wild red deer stags. Gen. Comp. Endocrinol..

[51] Clark MM, vom Saal FS, Galef BG (1992). Intrauterine positions and testosterone levels of adult male gerbils are correlated. Physiol. Behav..

[52] Kuijper EAM (2007). Heritability of reproductive hormones in adult male twins. Hum. Reprod..

[53] King RB, Cline JH, Hubbard CJ (2004). Heritable variation in testosterone levels in male garter snakes (*Thamnophis sirtalis*). J. Zool..

[54] Coviello AD (2011). Circulating testosterone and SHBG concentrations are heritable in women: The Framingham Heart Study. J. Clin. Endocrinol. Metab..

[55] Pavitt AT, Walling CA, Pemberton JM, Kruuk LEB (2014). Heritability and cross-sex genetic correlations of early-life circulating testosterone levels in a wild mammal. Biol. Lett..

[56] Ruth KS (2020). Using human genetics to understand the disease impacts of testosterone in men and women. Nat. Med..

[57] Fishman, R., Kralj-Fišer, S., Marglit, S., Koren, L. & Vortman, Y. Quantitative genetic study suggests sex-specific genetic architecture for fetal testosterone in a wild mammal. *bioRxiv* (2022). 10.1016/j.yhbeh.2024.105525 38452612

[58] Kilcoyne KR (2014). Fetal programming of adult Leydig cell function by androgenic effects on stem/progenitor cells. Proc. Natl. Acad. Sci. U. S. A..

[59] vom Saal FS, Bronson FH (1980). Sexual characteristics of adult female mice are correlated with their blood testosterone levels during prenatal development. Science.

[60] Ryan BC, Vandenbergh JG (2002). Intrauterine position effects. Neurosci. Biobehav. Rev..

[61] Beatty WW (1979). Gonadal hormones and sex differences in nonreproductive behaviors in rodents: Organizational and activational influences. Horm. Behav..

[62] Phoenix CH, Goy RW, Gerall AA, Young WC (1959). Organizing action of prenatally administered testosterone propionate on the tissues mediating mating behavior in the female guinea pig. Endocrinology.

[63] Dela Cruz C, Pereira OCM (2012). Prenatal testosterone supplementation alters puberty onset, aggressive behavior, and partner preference in adult male rats. J. Physiol. Sci..

[64] Edwards DA (1969). Early androgen stimulation and aggressive behavior in male and female mice. Physiol. Behav..

[65] vom Saal FS (1989). Sexual differentiation in litter-bearing mammals: Influence of sex of adjacent fetuses in utero. J. Anim. Sci..

[66] vom Saal FS, Bronson FH (1978). In utero proximity of female mouse fetuses to males: Effect on reproductive performance during later life. Biol. Reprod..

[67] Parfet KAR, Ganjam VK, Lamberson WR, Rieke AR (1990). Intrauterine position effects in female swine: Subsequent reproductive performance, and social and sexual behavior. Appl. Anim. Behav. Sci..

[68] Zielinski WJ, Saal FS, Vandenbergh JG (1992). The effect of intrauterine position on the survival, reproduction and home range size of female house mice. Behav. Ecol. Sociobiol..

[69] Meikle DB, Vessey SH, Drickamer LC, Meikle DB (1997). Testing models of adaptive adjustment of secondary sex ratio in domestic swine. Anim. Behav..

[70] Drickamer LC (1996). Intra-uterine position and anogenital distance in house mice: Consequences under field conditions. Anim. Behav..

[71] Clark MM, Malenfant SA, Winter DA, Galef BG (1990). Fetal uterine position affects copulation and scent marking by adult male gerbils. Physiol. Behav..

[72] Clemens LG, Gladue BA, Coniglio LP (1978). Prenatal endogenous androgenic influences on masculine sexual behavior and genital morphology in male and female rats. Horm. Behav..

[73] Houtsmuller EJ, Slob AK (1990). Masculinization and defeminization of female rats by males located caudally in the uterus. Physiol. Behav..

[74] Clark MM, Bishop AM, Vom Saal FS, Galef BG (1993). Responsiveness to testosterone of male gerbils from known intrauterine positions. Physiol. Behav..

[75] Clark MM, Tucker L, Galef BG (1992). Stud males and dud males: Intra-uterine position effects on the reproductive success of male gerbils. Anim. Behav..

[76] Clark MM, Galef BG (2000). Why some male Mongolian gerbils may help at the nest: Testosterone, asexuality and alloparenting. Anim. Behav..

[77] van der Hoeven T, Lefevre R, Mankes R (1992). Effects of intrauterine position on the hepatic microsomal polysubstrate monooxygenase and cytosolic glutathione S-transferase activity, plasma sex steroids and relative organ weights in adult male and female Long-Evans rats. J. Pharmacol. Exp. Ther..

[78] Borgia G, Blum MS, Blum NA (1979). Sexual selection and the evolution of mating systems. Sexual Selection and Reproductive Competition in Insects.

[79] Miller CW, Moore AJ (2007). A potential resolution to the lek paradox through indirect genetic effects. Proc. R. Soc. B Biol. Sci..

[80] Zambrano E, Guzmán C, Rodríguez-González GL, Durand-Carbajal M, Nathanielsz PW (2014). Fetal programming of sexual development and reproductive function. Mol. Cell. Endocrinol..

[81] Mills SC, Koskela E, Mappes T (2012). Intralocus sexual conflict for fitness: Sexually antagonistic alleles for testosterone. Proc. R. Soc. B Biol. Sci..

[82] Mokkonen M, Koskela E, Mappes T, Mills SC (2012). Sexual antagonism for testosterone maintains multiple mating behaviour. J. Anim. Ecol..

[83] Mokkonen M (2011). Negative frequency-dependent selection of sexually antagonistic alleles in *Myodes glareolus*. Science.

[84] Eales J, Thorpe RS, Malhotra A (2010). Colonization history and genetic diversity: Adaptive potential in early stage invasions. Mol. Ecol..

[85] Gao J (2019). Geographical and temporal variation of multiple paternity in invasive mosquitofish (*Gambusia holbrooki*, *Gambusia affinis*). Mol. Ecol..

