# Supplementary material for: Paternity share predicts sons’ fetal testosterone
Source: Sci Rep. 2023 Oct 4;13:16737. doi: 10.1038/s41598-023-42718-6 (PMC10551022; doi:10.1038/s41598-023-42718-6)
Supplement: Supplementary file 1 — Supplementary Information 1. [file 41598_2023_42718_MOESM1_ESM.pdf]

Supplemental Information A

Paternity share predicts sons' fetal testosterone

Ruth Fishman, Lee Koren, Rachel Ben-Shlomo, Uri Shanas, Yoni Vortman

Table SI-A1. Microsatellite primers used for assessing multiple paternity in 387 *Myocastor coypus* individuals (mothers and their fetuses).

The number of alleles (N_A), observed (H_O) and expected heterozygosity (H_E), and polymorphic information content (PIC). Loci that are not in Hardy-Weinberg equilibrium (HWE) are marked with an asterisk.

Locus	Primer sequence 5'-3'	N	N_A	H_O	H_E	PIC
McoD214	F: TTCACAAATCAGAGGCTACAATC<br>R: GTGTTCTTCATTTGATGCTCAG	385	10	0.865	0.823	0.798
McoA02	F: CCCACATGTATTTGCTTTTGAG<br>R: CAGATTCTGAGCACAGTGAGAC	385	5	0.657	0.648	0.613
McoD217	F: AGTCCAATCTACATAGCAAGGC<br>R: AGATAGCCCAGCAGAATAAGTG	366	3	0.454	0.448	0.398
McoD10*	F: TTTTGATACTAGCACCAATTATCTTTT<br>R: CTAAAGTTTGCAGCCTTGATTC	373	6	0.622	0.688	0.633
McoD60*	F: TCACTCAATAAATACTCAGGATGC<br>R: TGGGTGTAGGTGTAGGTAGACA	379	5	0.752	0.726	0.680
McoD215	F: GTTCAGACTTAGGAGTTGCTGG<br>R: GCTCCCTCGATACATTGATTAG	381	7	0.446	0.449	0.427
McoD69*	F: TTCCATCCCTGGTACCATATAC<br>R: TGAAGCATTAGATGCCTTTGTA	366	3	0.516	0.606	0.537
		Average	5.57	0.635	0.627	0.584

Table SI-A2. Distribution of multiple paternity in litters

Number of fathers	Number of litters
1	24
2	19
3	13
4	2
	Total: 58 litters
