# Supplementary material for: Paternity share predicts sons’ fetal testosterone
Source: Sci Rep. 2023 Oct 4;13:16737. doi: 10.1038/s41598-023-42718-6 (PMC10551022; doi:10.1038/s41598-023-42718-6)
Supplement: Supplementary file 2 — Supplementary Information 2. [file 41598_2023_42718_MOESM2_ESM.pdf]

Supplemental Information B

Paternity share predicts sons' fetal testosterone

Ruth Fishman, Lee Koren, Rachel Ben-Shlomo, Uri Shanas, Yoni Vortman

Examining 58 pregnant female carcasses allowed us to detect growth-retarded fetuses within the litter.

In our sample, we observed fourteen growth-retarded fetuses (10 males and 4 females; Table SI-B1 and Fig. SI-B1 a,b) from thirteen litters (estimated gestational ages of 52–126 days, of which 11 litters' gestational age was between 7-11 weeks, in accordance with Newson (1966) findings regarding the prevalent mortality age of individual fetuses). Six of these fetuses were assigned to a single father, seven to a main father, and only one fetus was assigned to a rare father. It is important to note that we examined the uterus in a specific time window, thus it is theoretically possible to miss potential future fetal absorptions.

Since in single-father pregnancies, only one option is possible – absorption of a fetus related to the single father, the analysis was performed on the multiple paternity litters. In these litters, only one of the 8 growth-retarded fetuses was related to a rare father (Table SI-B1). Notice, in this litter there was an additional fetus sired by the rare father, thus the number of fathers in this litter had not been reduced. **These results demonstrate that there is no mechanism to selectively absorb the fetuses sired by the rare father**. The resulting 'main father' potential fetal resorption has led to an increase in the ratio of fathers per litter size: a matched pair test for the difference in father's proportion in a litter in the multiple paternity litters before and after the subtraction of growth-retarded fetuses showed that the resulting higher proportion of fathers is significant (Wilcoxon Signed Rank, N=7; DF=6; P=0.0156, see Table SI-B1).

Table SI-B1. Conservation of number of fathers after potential resorption of growth-retarded fetuses

Litter ID	Fetus ID	Sex	Percent of litter's mean weight	Estimated pregnancy age (days)	N fathers	N fathers after potential resorption	Litter size	Litter size after potential resorption	Single/Main/Rare father of the growth retarded fetus
0014	0022	F	0.59	126	1	1	8	7	**Single**
0041	0046	M	0.22	58	1	1	7	6	**Single**
0083	0086	M	0.80	83	2	2	5	4	**Main**
0089	0093	F	0.73	57	2	2	8	7	**Main**
1008	1010	F	0.80	107	2	2	5	4	**Main**
0141	0142	F	0.60	62	3	3	5	4	**Main**
0156	0158	M	0.41	60	1	1	5	4	**Single**
0178	0183	M	0.36	66	2	2	7	6	**Rare***
0188	0192	M	0.57	57	1	1	5	4	**Single**
0212	0217	M	0.67	59	4	4	7	5	**Main**
0212	0219	M	0.79	59	4	4	7	5	**Main**
0236	0237	M	0.78	52	3	3	4	3	**Main**
0945	0952	M	0.07	62	1	1	7	6	**Single**
0229	0233	M	0.61	80	1	1	6	5	**Single**

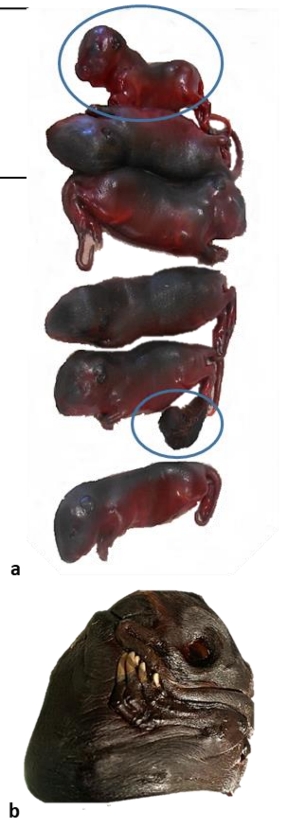

* Notice only one retarded growth fetus was sired by a rare father. Since there was more than one fetus sired by this rare father, the number of fathers in this litter has not declined, despite the potential resorption. While fetal absorption with respect to sire identity may be by chance, the increase in the ratio between number of fathers per litter size is significant, see text.

Figure SI-B1. Growth-retarded fetuses: (a) Litter containing two growth-retarded fetuses (circled). (b) A degenerative growth-retarded fetus.
